# A unique choline nitrate-based organo-aqueous electrolyte enables carbon/carbon supercapacitor operation in a wide temperature window (−40°C to 60°C)

**DOI:** 10.3389/fchem.2024.1377144

**Published:** 2024-04-11

**Authors:** Zhazira Supiyeva, Zulkhair Mansurov, Seitkhan Azat, Qamar Abbas

**Affiliations:** ^1^ Faculty of Chemistry and Chemical Technology, Al-Farabi Kazakh National University, Almaty, Kazakhstan; ^2^ Institute of Combustion Problems, Almaty, Kazakhstan; ^3^ Laboratory of Engineering Profile, Satbayev University, Almaty, Kazakhstan; ^4^ Faculty of Chemical Technology, Poznan University of Technology, Poznan, Poland; ^5^ Institute for Chemistry and Technology of Materials, Graz University of Technology, Graz, Austria

**Keywords:** anti-freezing electrolyte, supercapacitor, choline nitrate, methanol, low temperature, high temperature

## Abstract

Some drawbacks of aqueous electrolytes, such as freezing at low temperatures and extensive evaporation at high temperatures, restrict their industrial viability. This article introduces a stabilized neutral aqueous choline nitrate electrolyte with a 10 vol.% methanol additive that improves the temperature stability of the electrolyte via enhanced hydrogen bonding with the choline cation and water and maintains the good state of health of the supercapacitor cells under extreme operating conditions. The symmetric carbon/carbon supercapacitor in 5 mol/kg choline nitrate + 10 vol.% methanol (σ = 76 ms/cm at 25°C) exhibits 103 F/g at room temperature during galvanostatic charge/discharge up to 1.5 V, which decreases to 78 F/g at −40°C due to the suppressed Faradaic reactions occurring at the carbon electrode. However, under similar charge/discharge conditions, the capacitance increases to 112 F/g when the supercapacitor operates at 60°C. This capacitance increase at high temperatures is due to the Faradaic reactions related to enhanced hydrogen adsorption and desorption. The most remarkable aspect of the proposed supercapacitor is its ability to maintain capacitance and power performance during high voltage floating at 1.5 V at three tested temperatures (−40°C, 24°C, and 60°C).

## 1 Introduction

Electrochemical capacitors, also known as supercapacitors, use activated carbon-based electrodes possessing a high surface area (up to 2,500 m^2^/g) in organic or aqueous electrolytes (Schranger et al., 2020; Liu Ch. et al., 2021; Yakaboylu et al., 2021). Due to the physical charge storage at the electrical double layer (EDL) developed in the nanopores of carbon material, the adsorption and desorption of ions are quite fast, giving high power characteristics to supercapacitors with a time constant τ < ms. The temperature-related performance of the supercapacitor is determined by the freezing and evaporation behavior of the electrolyte, and therefore, organic electrolytes are generally preferred in commercial supercapacitors due to the low vapor pressure of the solvent (Burke, 2000; Burke and Miller, 2010). Nevertheless, organic electrolytes are expensive and produced with hazardous acetonitrile solvents. Supercapacitors using water-based electrolytes can operate in a limited temperature window due to the high water vapor pressure (Béguin et al., 2014; Babu et al., 2020). In particular, the neutral aqueous electrolytes, which exhibit high voltages up to 1.6–1.8 V, are interesting (Raymundo-Piñero et al., 2006; Bichat et al., 2010; Demarconnay et al., 2010; Gao et al., 2012; Abbas et al., 2014; Abbas et al., 2020; Galek et al., 2020; Supiyeva et al., 2023). However, the presence of water as a solvation source restricts their low- and high-temperature applications.

At low temperatures, a water/ethylene glycol mixture has been tested in carbon/MnO_2_ asymmetric supercapacitors, which operate down to −30°C (Roberts et al., 2013). Carbon/carbon supercapacitors using aqueous Li_2_SO_4_ electrolyte in a water/methanol mixture have been tested to −40°C (Abbas and Béguin, 2016), where the influence of carbon pore size on the freezing behavior of the solvent mixture at different volume ratios has been investigated. It was found that water adsorbed, preferably into the carbon pores, leaving behind a high local concentration of methanol that prevents the freezing of bulk electrolytes at low temperatures. The supercapacitor exhibited high capacitance and Coulombic efficiency at room temperature, which decreased greatly at −40°C due to reduced contribution from Faradaic processes at low temperatures; however, both Coulombic and energy efficiency values increased with decreasing temperatures. In order to eliminate the issue of low Li_2_SO_4_ solubility in a water/methanol mixture and consequently low electrolyte conductivity, an aqueous solution of choline nitrate was proposed to work at low temperatures of ca. −40°C in both a carbon/carbon setup (Abbas and Béguin, 2018) as well as a carbon/MnO_2_ system (Schrade et al., 2022). In the carbon/carbon system case, positive electrode degradation was observed during potentiostatic floating when operating at 24°C and −40°C. The issue of positive electrode degradation was then tackled by replacing it with a relatively more stable MnO_2_ positive electrode in the latter case (Schrade et al., 2022). Other strategies for achieving a low-temperature performance of supercapacitors include applying various electrolyte compositions in solid-state systems (Rong et al., 2018; Liu J. et al., 2021; Zhang et al., 2021; Tu et al., 2022), up to 30% glycerol in electrolytes (Railanmaa et al., 2019), or hydrogel electrolytes (Zeng et al., 2023; Zhang et al., 2023; Nan et al., 2024).

High temperatures, on the other hand, strongly influence the electrolyte properties and the performance of electrodes at both the positive and negative terminals. The positive electrode can undergo irreversible redox reactions (Vellacheri et al., 2014; Xiong et al., 2015), while hydrogen evolution can occur at the negative electrode of an aqueous electrode (Galinski et al., 2013). The carbon electrode oxidation in supercapacitors using aqueous electrolytes has been widely discussed, and the presence of surface functional groups such as carboxylic acid, quinone, carbonyl, or ketones has been reported (Fan et al., 2013; Kerisit et al., 2014; Cao et al., 2018; He et al., 2018; Yang et al., 2018; Ilnicka et al., 2021; Qiu et al., 2023). Carbon electrodes are highly reactive due to the presence of dangling bonds that are developed as a result of free electrons present at the edge sites (Enoki et al., 1994; Manivannan et al., 1999; Kiguchi et al., 2011; Eleri et al., 2020). While the low-temperature operation of supercapacitors suppresses the onset of these reactions and, consequently, the evolution of the functional groups on the electrode surface, high temperatures favor the formation of surface functional groups due to the low activation energy needed for the chemical reaction. In addition, the aqueous electrolytes have strong corrosive properties toward current-collector materials at elevated temperatures, another drawback restricting their applicability.

It was particularly important to find electrolyte compositions that favor the high-temperature optimal performance of supercapacitors in aqueous electrolytes while minimizing the risk of surface functional group generation in order to improve the performance of the device. In this regard, several additives have been used in water-based electrolytes for zinc-ion batteries, lithium-ion cells, hybrid supercapacitors, and symmetric supercapacitors. Methanol has been used as an additive that promotes a hydrogen bonding network within the solvent molecules, thus decreasing the vapor pressure effect when used in supercapacitor cells. In addition, the eutectic effect of choline nitrate in water is promoted by the addition of methanol, which directly interacts with the choline cation and the nitrate anion. The presence of enhanced hydrogen bonding due to the –OH groups in water and methanol stabilizes the ionic interactions between solvent molecules and with the ionic species in the electrolyte.

In this work, we used a choline nitrate-based electrolyte in water with 10 vol.% methanol as an additive for a carbon/carbon symmetric supercapacitor that operates in a wide temperature window of −40°C to 60°C. The capacitance, energy efficiency, and Coulombic efficiency values were estimated and compared at these temperatures. Furthermore, physicochemical techniques such as thermogravimetric analysis, Raman spectroscopy, and electron microscopy were used to characterize the electrode and electrolyte materials. Overall, it was found that high temperatures have a worse effect on a carbon electrode than low temperatures. Meanwhile, the addition of methanol protected the positive carbon electrode from oxidation. In addition, hydrogen evolution at the negative electrode was reduced at low temperatures due to thermodynamic limitations and at high temperatures due to the promoted hydrogen bonding between the water solvent and methanol as an additive.

## 2 Experimental setup

### 2.1 Electrolyte preparation and characterization

Choline nitrate (ChNO_3_) was produced from choline chloride (ChCl) through a metathesis reaction by exchanging chloride with nitrate, according to the procedure described in the literature (Curnow et al., 2018; Abbas et al., 2021). The electrolyte solution was prepared with a concentration of 5 mol/kg by dissolving the weighed amount of salt in distilled water. The salt was mixed under continuous stirring at room temperature, and 10 vol.% methanol was gradually poured into the solution. The final electrolyte solution, 5 mol/kg choline nitrate + 10 vol.% methanol, was characterized as pH = 6.9, conductivity 76 ms/cm, and viscosity 2.25 mPa s at 24°C. When the proportion of methanol was increased to 20 vol.%, the electrolyte ionic conductivity decreased to 51 ms/cm. Therefore, 5 mol/kg choline nitrate + 10 vol.% methanol was selected as the appropriate composition for electrochemical studies. The conductivity of the electrolyte was determined using a YSI 3200 Laboratory conductivity meter (USA), the viscosity using an A&D Weighing SV-1A SV-A Series Sine-wave vibro viscometer (Japan), and Raman spectroscopy measurements were performed with the scanning probe “Ntegra Spectra” using a 632.8 nm laser.

### 2.2 Electrode preparation and characterization

Carbon-sheet electrodes were used to assemble the supercapacitors. The electrode sheet was made of 90 wt.% of YP80 F carbon (from Kuraray, Japan), 5 wt.% of SUPER C65 carbon black (Imerys) as a conductivity enhancer, and 5 wt.% of polytetrafluoroethylene (60 wt.% suspension in water, Sigma-Aldrich) as a binder. These three components were mixed in isopropanol solvent and stirred at 70°C until a dough was obtained; then the dough was rolled to produce an ∼ 80 μm thick sheet. The carbon sheet was dried at 100°C overnight and cut into small disks of diameter 1.0 cm. Symmetric Swagelok-type supercapacitors were assembled by sandwiching electrodes (approximately 3.0 mg each) separated by a glass microfiber separator (Whatman GF/A, 260 μm thick, diameter = 1.2 cm). In order to monitor the potential profile and other performance characteristics of individual electrodes, a two-electrode setup was equipped with a reference electrode (in this case, an Ag/AgCl reference electrode in 3 mol/L KCl) while maintaining the same mass of active material in two electrodes. All electrochemical testing of the supercapacitor cells was performed using a multi-channel VSP potentiostat (BioLogic, France), while the cells were placed in a climatic chamber.

### 2.3 Electrochemical testing

The supercapacitor cells in the two-electrode setup were assembled using the procedure given above and tested using cyclic voltammetry and galvanostatic charge/discharge methods. The long-term stability was determined by constant voltage hold tests (also known as a floating test). A total hold period of 100 h was selected, during which capacitance and resistance values were determined at specific intervals. In the case of three-electrode measurements for hydrogen evolution studies, an oversized counter electrode was used by keeping a working electrode to counter electrode mass ratio of 1:5. While the reference electrode was the same as described in Section 2.2, two separators were used to maintain access to the electrolyte during electrochemical polarization. The working electrode potential was varied in a wide negative window to monitor the hydrogen evolution characteristics.

### 2.4 Postmortem analysis of electrodes

Fresh carbon electrodes were investigated by scanning electron microscopy (SEM) on a Quanta 3D 200i Dual System microscope (FEI, United States) with an accelerating voltage of 30 kV, Energy Dispersive X-ray (EDX), and specific area mapping to observe any metals or other elements as impurities. Raman spectroscopy was conducted before and after the electrochemical testing and potentiostatic cycling at high voltage, and the changes in D- and G-bands were monitored. Thermogravimetric (TG) and differential thermal analysis (DTA) tests were carried out via a TA Instruments Q600 Simultaneous DSC/TG, which measures the weight change in a material as a function of temperature or time under a controlled atmosphere. For the TGA *postmortem* analysis of carbon electrodes after aging tests at 60°C, samples were heated from room temperature to 700°C at a rate of 10°C min^−1^.

## 3 Results and discussion

### 3.1 Preparation and investigation of electrodes and electrolytes

**FIGURE 1:**
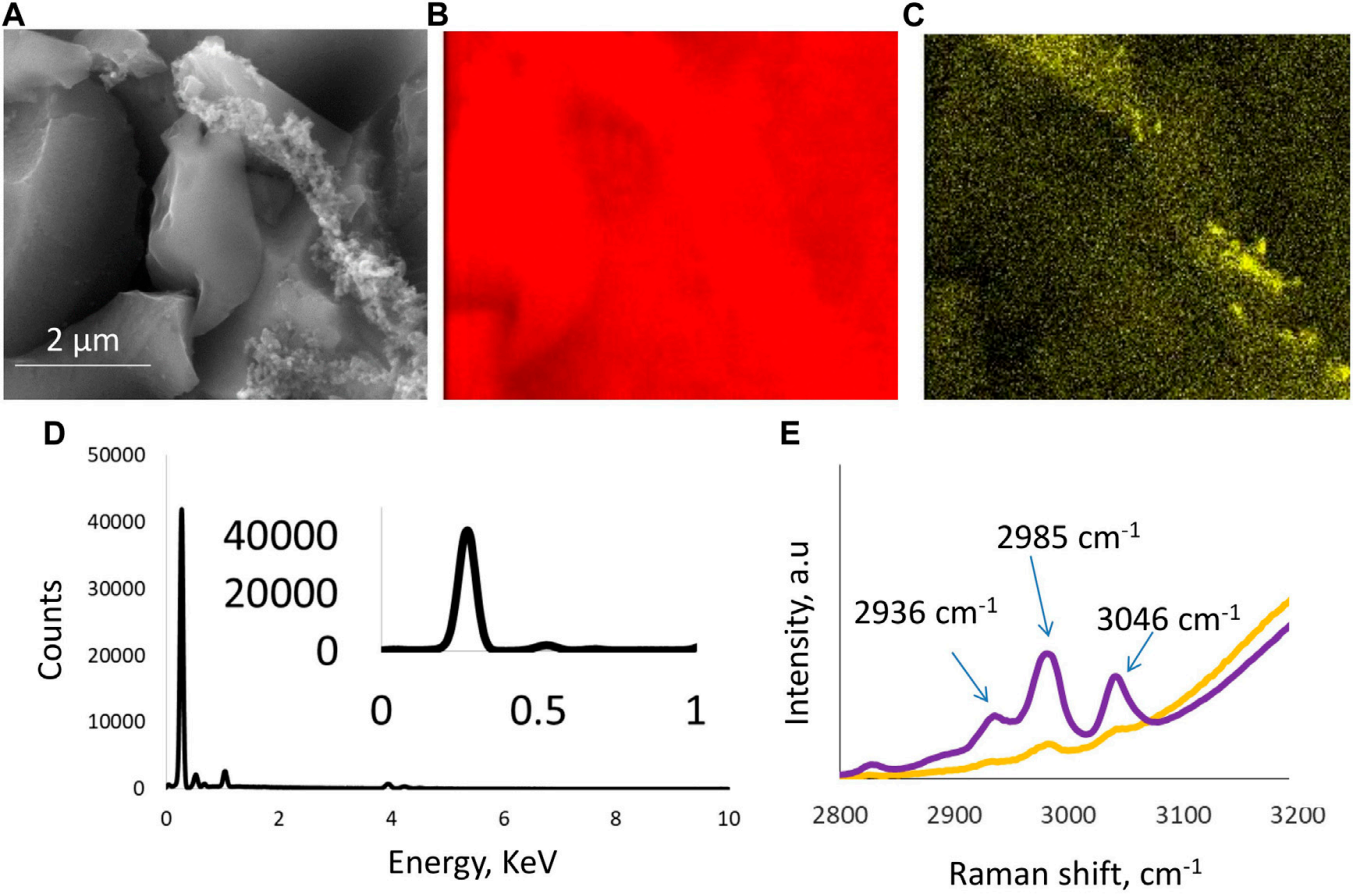
Physicochemical characterization of the electrode and the electrolyte: **(A)** SEM image of a carbon electrode where carbon particles are visible and bound together with PTFE binder; **(B,C)** also confirmed by the area mapping micrographs; **(D)** EDX analysis of a carbon electrode; and **(E)** Raman spectra of aqueous electrolyte 5 mol/kg choline nitrate without (yellow curve) and with (purple curve) 10 vol.% methanol.

The morphology of the freshly produced electrodes was monitored by SEM and EDX analysis. Specific area mapping by SEM indicated the presence of carbon as the main component in the electrode, along with a binder that was only visible in selected regions of the electrode. Figures 1A–C show the presence of large particles of the activated carbon material used to produce electrodes that are interconnected by the presence of PTFE polymeric structures. The electrolyte was 5 mol/kg choline nitrate + 10 vol.% methanol at pH 6.7, and it maintained this value over a wide temperature range from −10°C to 40°C, whereas the ionic conductivity at room temperature was 76 ms/cm. Figure 1D shows an EDX analysis of a carbon electrode in which carbon was detected as the main element. The Raman spectra in Figure 1E present a comparison between the two solutions, one with and one without the presence of methanol. Raman peak evolution at 3,046 cm^−1^, 2,985 cm^−1^, and 2,936 cm^−1^ indicates a range of C-H and O-H stretching bands. This bonding can be attributed to the presence of a strong hydrogen bond between the –OH groups of methanol and the –CH_2_ groups present in the choline structure. Another contribution to these peaks evolving with the addition of methanol can be attributed to the hydrogen bond developed between water and methanol molecules and the –OH groups of the choline cation. These findings suggest strong molecular interactions between choline nitrate, methanol, and water, that could influence various physicochemical and electrochemical properties of the electrolyte and, consequently, affect its performance during electrochemical testing. Hence, our work reveals for the first time the stabilization of an aqueous choline-based electrolyte due to hydrogen bonding as elaborated by Raman spectroscopy peak analysis.

### 3.2 Hydrogen adsorption/desorption investigations

**FIGURE 2:**
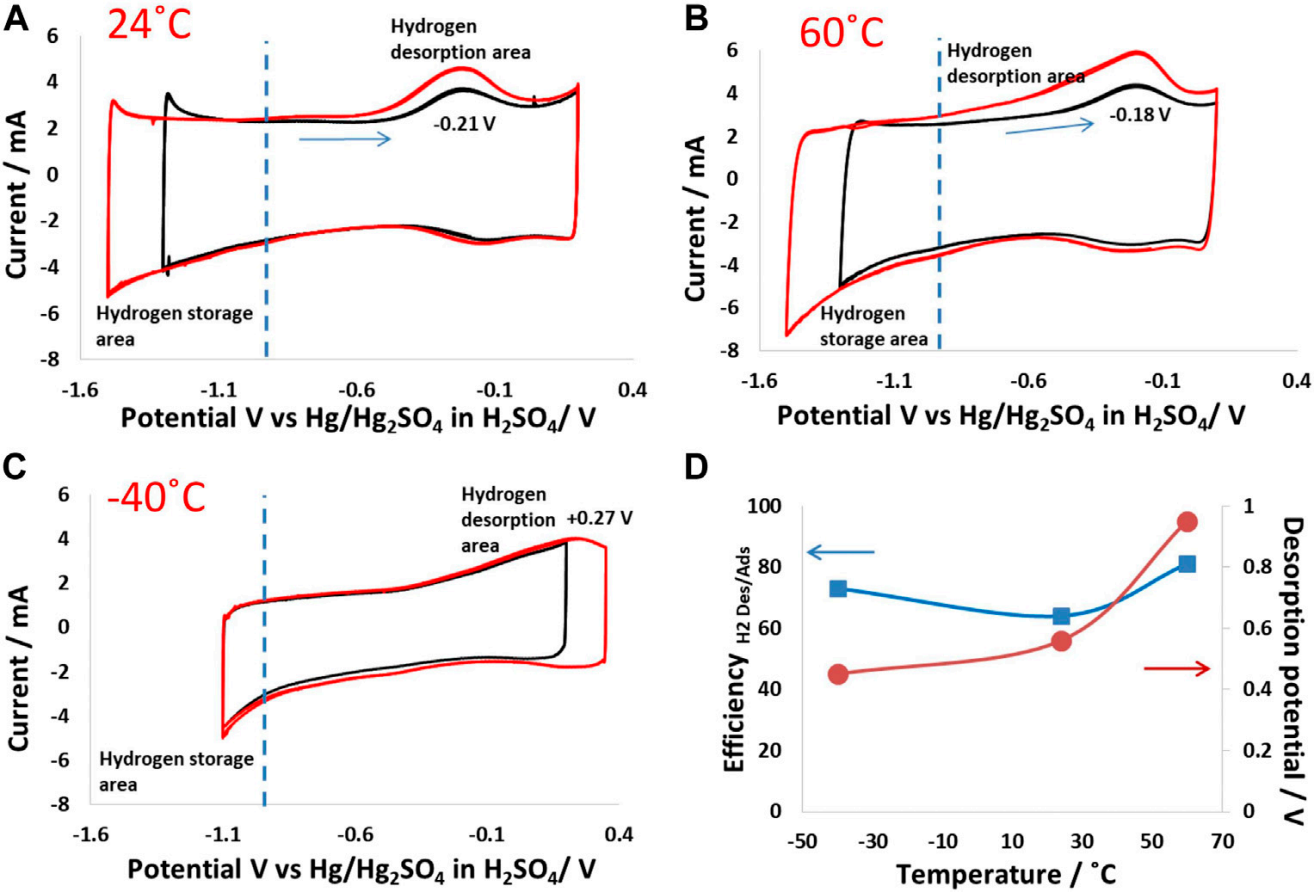
Hydrogen adsorption/desorption on a carbon electrode (same electrode mass was maintained in all experiments): electrochemical polarization of the porous carbon electrode in 5 mol/kg choline nitrate + 10 vol.% methanol from 0.2 V to −1.5 V vs. Hg_2_SO_4_ in H_2_SO_4_ at **(A)** 24°C; **(B)** 60°C; and **(C)** −40°C; **(D)** graph showing the energy efficiency of hydrogen adsorption/desorption and potentials vs. implemented temperature.

Hydrogen adsorption and desorption were studied via electrochemical polarization of a porous carbon electrode in 5 mol/kg choline nitrate + 10 vol.% methanol in a wide potential window. The potential on the positive side was fixed, while the potential value in the negative region was changed step-wise, where electrochemical reduction of water takes place to produce nascent hydrogen and OH^−^ ions. While the nascent hydrogen participates in chemical bonding with the carbon electrode (Chun and Whitacre, 2013; Abbas et al., 2015), OH^−^ ion generation influenced the local pH at the electrode surface, leading to a rise in the local pH value in the carbon pores. Figure 2A shows the hydrogen adsorption/desorption behavior of the carbon electrode at 24°C. In this case, the positive potential was fixed at 0.2 V vs. ref., and the negative cutoff potentials were fixed at −1.3 V vs. ref. or −1.5 V vs. ref. As the potential was reduced to −1.2 V, an increase in negative current indicated an enhanced electrochemical water reduction, which further increased as the carbon electrode was polarized to −1.5 V. Simultaneously, upon a reverse scan toward positive potentials, humps at −0.2 V related to hydrogen desorption from the carbon electrode could be seen. The energy efficiency for hydrogen adsorption/desorption is ∼78%, estimated by dividing the integrated area of desorption by the integrated area of adsorption.

Figure 2B shows the hydrogen evolution behavior at 60°C, where high hydrogen adsorption can be seen as a higher negative current that was reached for a similar cutoff potential than at 24°C. Nevertheless, the desorption peak at 60°C was broad, which indicates the chemical bonding of hydrogen with carbon possessing different energies. This could be because hydrogen was adsorbed at different sites within the carbon electrode matrix, ranging from the surface of the particle to the inner pore matrix. In addition, the desorption at a wide potential window also indicates a range of chemical bonding from weak to strong, depending on the adsorption kinetics, which are strongly influenced by temperature. Indeed, the desorption peak for the polarization with a cutoff potential of 1.5 V vs. Hg_2_SO_4_ in H_2_SO_4_ was much broader than at 1.2 V, which shows that the nature of hydrogen bonded to carbon was strongly influenced by the electrical potential applied for the production of nascent hydrogen. It is important to mention that the forward scan, where the production and adsorption of hydrogen took place, did not show multiple peaks, which confirms the single electro-reduction step of water and simultaneous hydrogen production and chemical bonding at feasible sites in the carbon electrode matrix.

Compared to the foregoing cases, the hydrogen production and adsorption mechanisms were strongly influenced at low temperatures. Figure 2C shows cyclic voltammograms at −40°C, polarized between 0.3 V and −1.1 V vs. Hg_2_SO_4_ in H_2_SO_4_. Hydrogen formation was suppressed due to the quenching of Faradaic reactions, which are thermodynamically unfavorable at such low temperatures. The water reduction was decreased at −40°C, meaning that the charge was mainly stored at the EDL of the electrode. It could also be seen that the desorption peaks were weak compared to the cases at 24°C and 60°C. In addition, the desorption potential at −40°C was shifted to high positive values, indicating that it was thermodynamically difficult to extract hydrogen adsorbed at low temperatures. Thus, the main charge storage phenomenon at −40°C was EDL, and only a small amount of hydrogen was adsorbed, which could then be desorbed, indicating high energy efficiency, as shown in Figure 2D. Overall, the operating temperature strongly influenced the hydrogen adsorption/desorption characteristics produced due to the electrochemical reduction of water in the electrolyte.

### 3.3 Supercapacitor performance at room temperature

**FIGURE 3:**
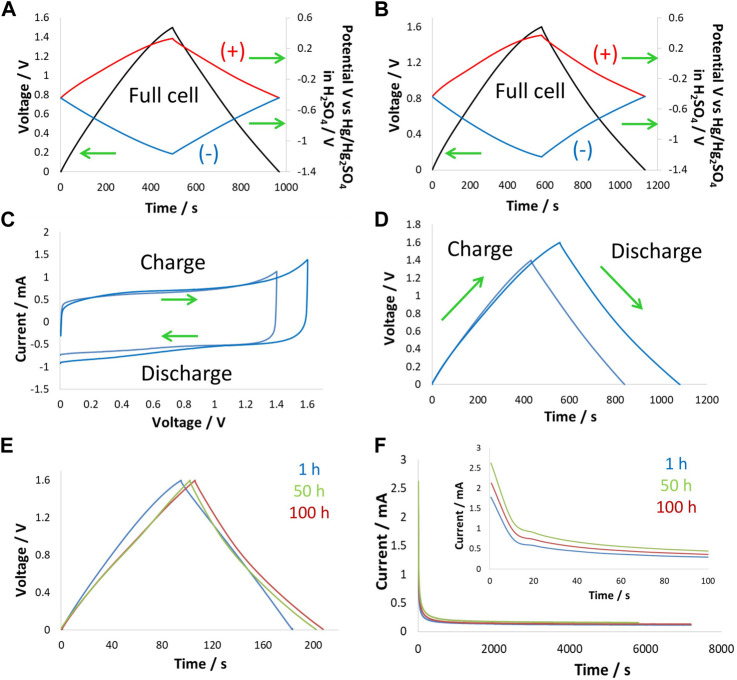
Electrochemistry at 24°C: two-electrode cell with a reference electrode in 5 mol/kg choline nitrate + 10 vol.% methanol polarized at 200 mA/g to **(A)** 1.5 V and **(B)** 1.6 V; **(C)** CV at 2 mVs; and **(D)** GCPL curves at 200 mA/g to 1.5 V and 1.6 V obtained in a two-electrode setup, **(E)** GCPL curves at 200 mA/g during floating at 1.6 V after 1 h, 50 h, and 100 h, and **(F)** leakage current profiles obtained during floating at 1 h, 50 h, and 100 h.

Potentially limited galvanostatic charge/discharge (g*alvanostatic cycling* with potential limitation, GCPL) curves for a two-electrode supercapacitor up to 1.6 V at 24°C showed symmetric behavior. This cell was equipped with a reference electrode to monitor positive and negative electrode potential profiles (Figures 3A, B). The positive electrode at a cell voltage of 1.5 V operates within a potential window of 0.75 V, meaning that the potential window is nearly equally divided between the two electrodes at this voltage. When polarizing the cell to a voltage of 1.6 V, the positive electrode potential only increased by 20 mV (0.77 V), while the negative electrode operated with a potential of 0.83 V. The positive electrode at the cell voltage of 1.6 V reached the oxidation potential of water, which was the reason for the shortening of its potential window. The effect of water oxidation both shortened the potential window of the positive electrode and slightly disturbed the symmetric charge/discharge behavior of the two electrodes. The impact of this effect can be seen in the overall energy efficiency of the cell, which decreased from 91% at 1.5 V to 78% at 1.6 V.

CV and GCPL curves obtained on two-electrode supercapacitor cells with the same electrolyte shown in Figures 3C, D, respectively, confirmed the previous findings. Here, one can see the increased current on the CV and asymmetry of the GCPL curves when increasing voltage from 1.5 V to 1.6 V. Furthermore, these supercapacitor cells were subjected to potentiostatic floating (voltage hold test) at 1.6 V for 100 h, during which GCPL curves and leakage current profiles after 1 h, 50 h, and 100 h were collected. Figure 3E shows the varying shape of GCPL curves during floating, which suggests that the supercapacitor’s state of health was strongly impacted by the voltage hold test. The energy efficiency of the two-electrode cell was ∼90% after the first hour of floating and decreased to 76% after 50 h of floating and to 73% after 100 h of floating. It appears that the strong impact of floating occurred at the beginning of the test and then stabilized as the electrode surface was passivated by the presence of surface functional groups. Figure 3F shows that upon floating, the Faradaic reactions started to appear, which caused an increase in the leakage current, whereas, after an initial increase (after 50 h), the profile stabilized and did not further increase up to 100 h of floating.

**FIGURE 4:**
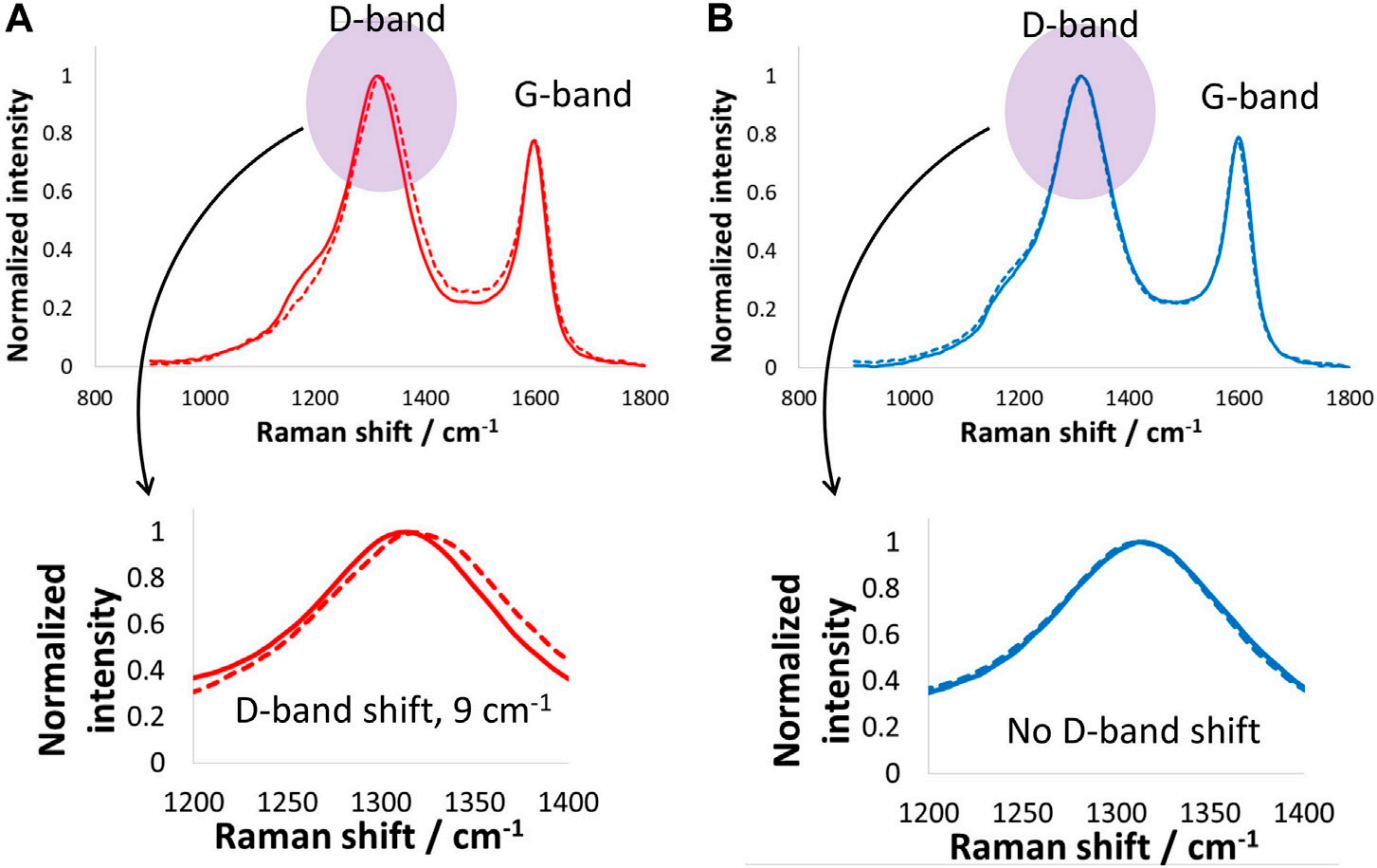
Raman spectroscopy investigations (D- and G-band) of **(A)** positive and **(B)** negative carbon electrodes after electrochemical tests and floating of the supercapacitor cell at 1.6 V for 100 h at room temperature.

Positive and negative carbon electrodes extracted from the above-mentioned supercapacitor were subjected to Raman spectroscopy measurements (Figure 4), and the D-band and G-band shifts were specifically monitored during these tests. The positive electrode exhibited a D-band upshift of 9 cm^−1^, while the negative electrode did not show any changes in the D-band position. On the other hand, the G-band in both the positive and negative electrodes remained unchanged. No shift in the G-band confirms no structural lattice changes occurred in the carbon electrode; however, the large upshift of the D-band in the Raman spectrum of the positive electrode suggests surface changes taking place during harsh floating tests. As explained above regarding the change in the GCPL curve when increasing cell voltage, most of the changes recorded for the positive electrode are due to the oxygenated surface functional groups generated during supercapacitor operation at high voltage.

### 3.4 Supercapacitor testing at low temperatures (−40°C)

**FIGURE 5:**
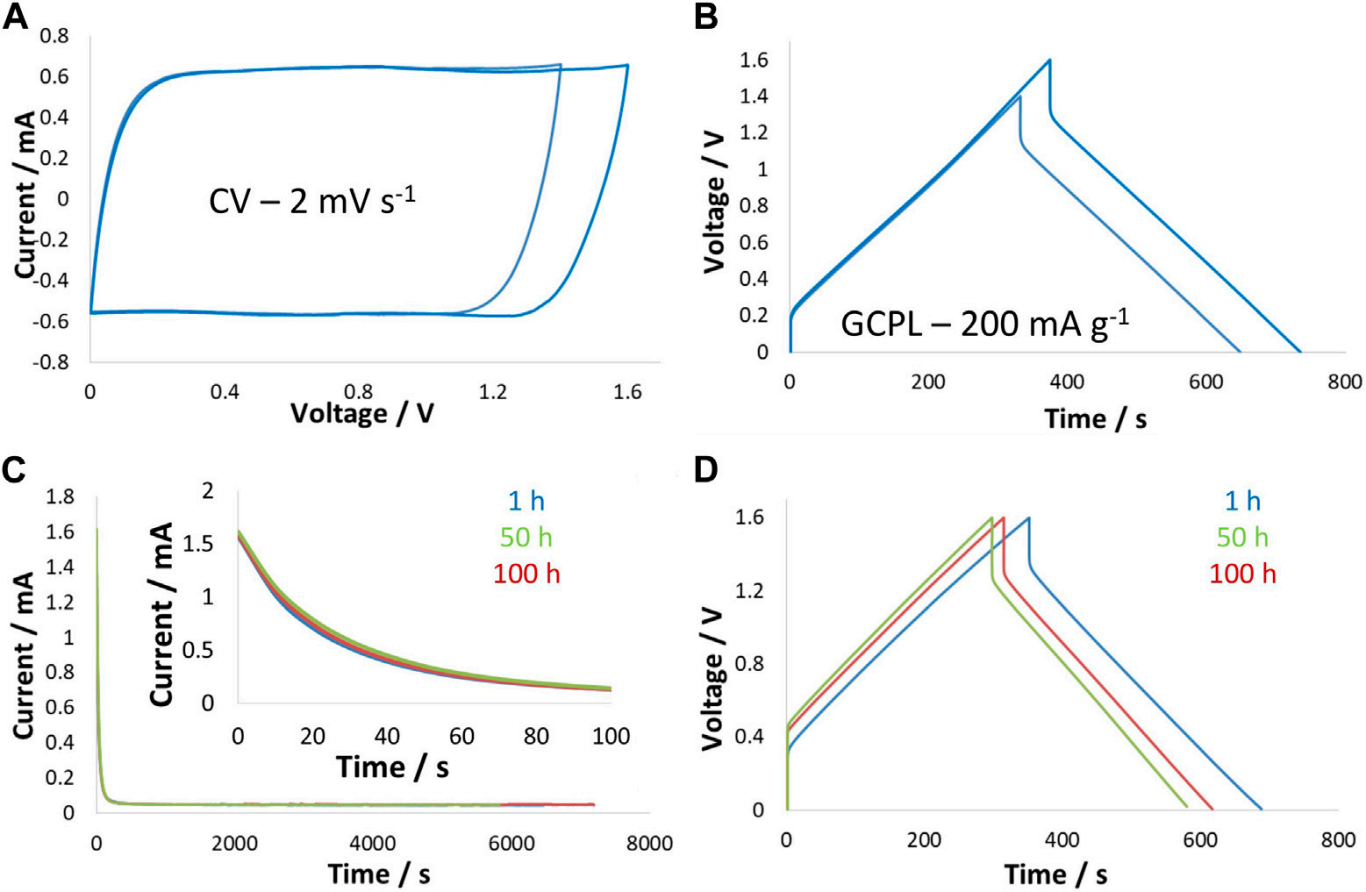
Performance of the two-electrode supercapacitor cell in 5 mol/kg choline nitrate + 10 vol.% methanol at −40°C: **(A)** CVs at 2 mV/s; and **(B)** GCPL curves at 200 mA/g up to 1.5 V and 1.6 V; **(C)** leakage current profiles during floating at 1 h, 50 h, and 100 h; and **(D)** GCPL curves obtained after 1 h, 50 h, and 100 h of floating at 1.6 V.

The electrochemical performance of a supercapacitor cell at −40°C is presented in Figure 5. CVs and GCPL curves up to 1.6 V were recorded at 2 mV/s and 200 mA/g, respectively. The CV curves show square-shaped characteristics that are typical of EDL charge storage. Compared to the two-electrode CVs in Figure 3C, the current increase was absent when reaching a high voltage of 1.6 V, which is due to the suppression of Faradaic processes at low temperatures. Particularly, the Faradaic reactions related to hydrogen evolution, which were thermodynamically unfavorable, were eliminated at −40°C. Therefore, the symmetric charge/discharge at such a low temperature and the high energy efficiency of 97% can also be explained by the absence of Faradaic reactions—no additional charge was consumed. Nevertheless, the high ohmic drop at −40°C was obviously visible and can be attributed to a few factors. First, bulk electrolyte conductivity decreased enormously at low temperatures (σ_24°C_ = 76 ms/cm compared to σ_0°C_ = 39 ms/cm), which caused concentration gradient-related resistance. Another factor contributing to the high ohmic drop at low temperatures was the impact on ionic transport within the carbon nanopores. Freezing of electrolytes against the pore wall is a well-known phenomenon that can give rise to high ohmic loss. This effect can be confirmed by the viscosity values, which increased from 2.13 mPa s (at 24°C) to 4.14 mPa s (at −10°C). Overall, such a high ohmic drop at low temperatures resulted in reduced power performance for the supercapacitor.

Results of the potentiostatic floating performed at 1.6 V at −40°C show an unchanged state of health of the supercapacitor due to the absence of Faradaic reactions and no generation of surface functional groups. This was confirmed by the unchanged leakage profiles collected after 1 h, 50 h, and 100 h of floating at 1.6 V. Figure 5D presents a comparison of the charge/discharge curves up to 1.6 V during floating, and the profile of these curves remained nearly unchanged while the capacitance decreased, which could be due to the partial freezing of the electrolyte in the electrode pores leading to pore blockage.

**FIGURE 6:**
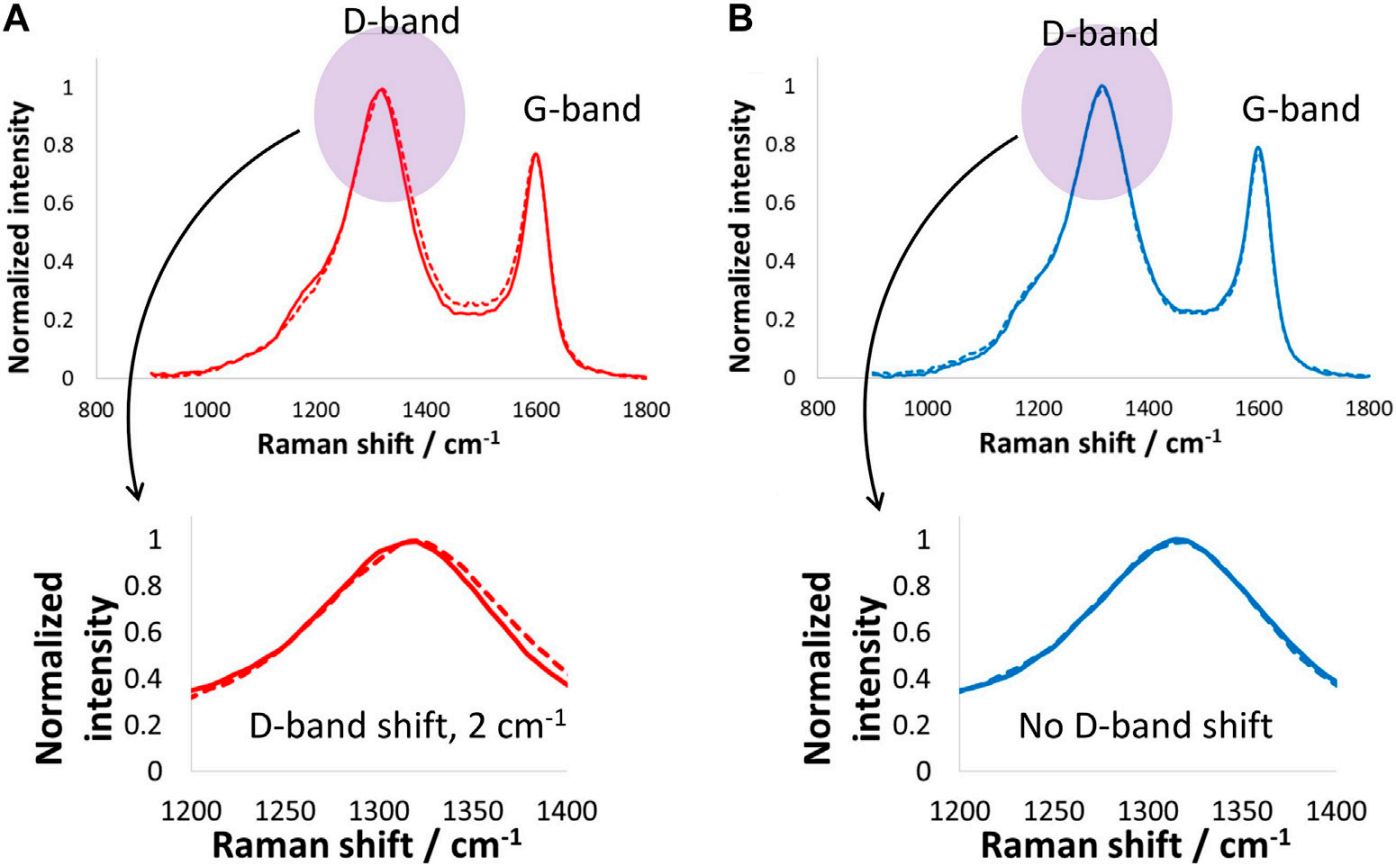
Raman spectroscopy investigations (D- and G-band) of **(A)** positive and **(B)** negative carbon electrodes after electrochemical tests and floating of the supercapacitor cell at 1.6 V for 100 h at −40°C.

Figure 6 shows the Raman spectra of the positive and negative electrodes after the floating tests performed at 1.6 V for 100 h. The positive electrode Raman spectrum shows that the D-band upshifted by only 2 cm^−1^, which suggests the floating did not impact the carbon electrode surface at such low temperatures, mainly due to the absence of Faradaic reactions. On the other hand, the negative electrode D- and G-bands remained unchanged, which suggests that the carbon material on the negative electrode was not influenced by the floating. Overall, both the positive and negative electrodes maintained a good state of health while floating at 1.6 V and −40°C.

### 3.5 Supercapacitor performance at high temperatures (60°C)

**FIGURE 7:**
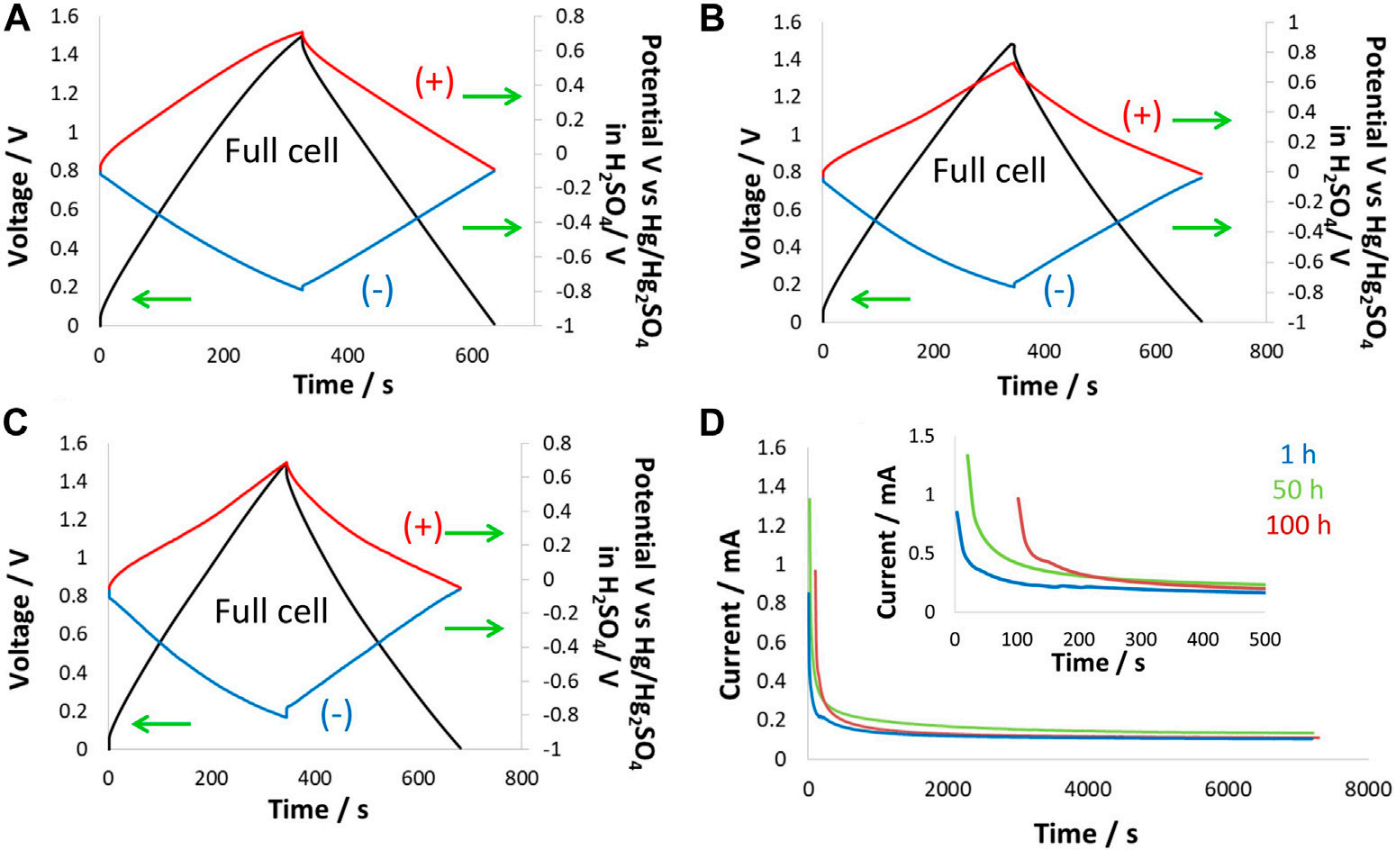
Electrochemical testing at 60°C: two-electrode cell equipped with the reference electrode in 5 mol/kg choline nitrate + 10 vol.% methanol was tested during floating at 1.5 V for 100 h, **(A)** GCPL curves at 200 mA/g up to 1.5 V after 1 h; **(B)** 50 h and **(C)** 100 h of floating; and **(D)** leakage current profiles collected during floating for 1 h, 50 h, and 100 h.

Supercapacitor cells were tested at 1.5 V at 60°C to observe the effect of high temperature on the performance of supercapacitors in a choline nitrate + methanol-based electrolyte. Because temperature greatly influences electrolyte degradation and affects the performance of electrode material, cells were equipped with a reference electrode to monitor the individual electrode. Figure 7 shows the GCPL curves of cells and electrodes collected at 200 mA/g during potentiostatic floating tests at 1.5 V for 100 h. At the beginning of floating, the GCPL curve for the supercapacitor cell was symmetric, while the positive and negative electrodes in Figure 7A operated in nearly similar potential windows. The positive electrode potential window of 0.76 V and the negative electrode potential window of 0.74 V were in close agreement and divided a nearly equal share of the potential window for the cell. The overall cell energy efficiency was found to be 92%, which is quite high given the fact that the cell was operating at 60°C. After 50 h of floating at 1.5 V, the GCPL curves for the cell were slightly deformed, with the main contribution coming from the positive electrode, which was more influenced at high temperatures than the negative electrode. It is understood that the positive electrode was operating near the electrolyte oxidation potential limit. Thus, combining this factor with the high temperature at which aqueous electrolyte activity was high, the positive electrode underwent relatively more degradation than the negative electrode. As discussed in previous studies, the negative electrode was not substantially influenced during the floating test due to the electro-reduction of water causing hydrogen adsorption/desorption, which is a highly reversible process at the nanoporous carbon electrode.

After 100 h of floating at 1.5 V and 60°C, Figure 7C shows that the GCPL curve for the supercapacitor cell was substantially deformed, and the main contribution came from the positive electrode, which exhibited a narrow GCPL profile toward high positive potentials. This effect on the carbon electrode has been described as pore blockage due to the generation of oxygenated surface functional groups that affect the positive electrode. On the other hand, the negative electrode, even after 100 h of floating, was not much influenced; the energy efficiency was observed to be still very high, ∼67%. Leakage current profiles in Figure 7D show the temperature effects that caused high leakage current at the beginning of the floating period dedicated to the reorganization of charges within the carbon porosity. The leakage current increased over floating time and during steady-state conditions, which confirms the dual effect of temperature and high-voltage operation. Overall, a comparison of leakage current profiles for the first 100 s showed a slight increase at room temperature from 0.16 to 0.18 mA while remaining nearly constant at −40°C. However, at the high temperature of 60°C, the leakage current increased enormously from 0.24 to 0.90 mA during the period of 100 h of floating due to the onset of a Faradaic reaction.

**FIGURE 8:**
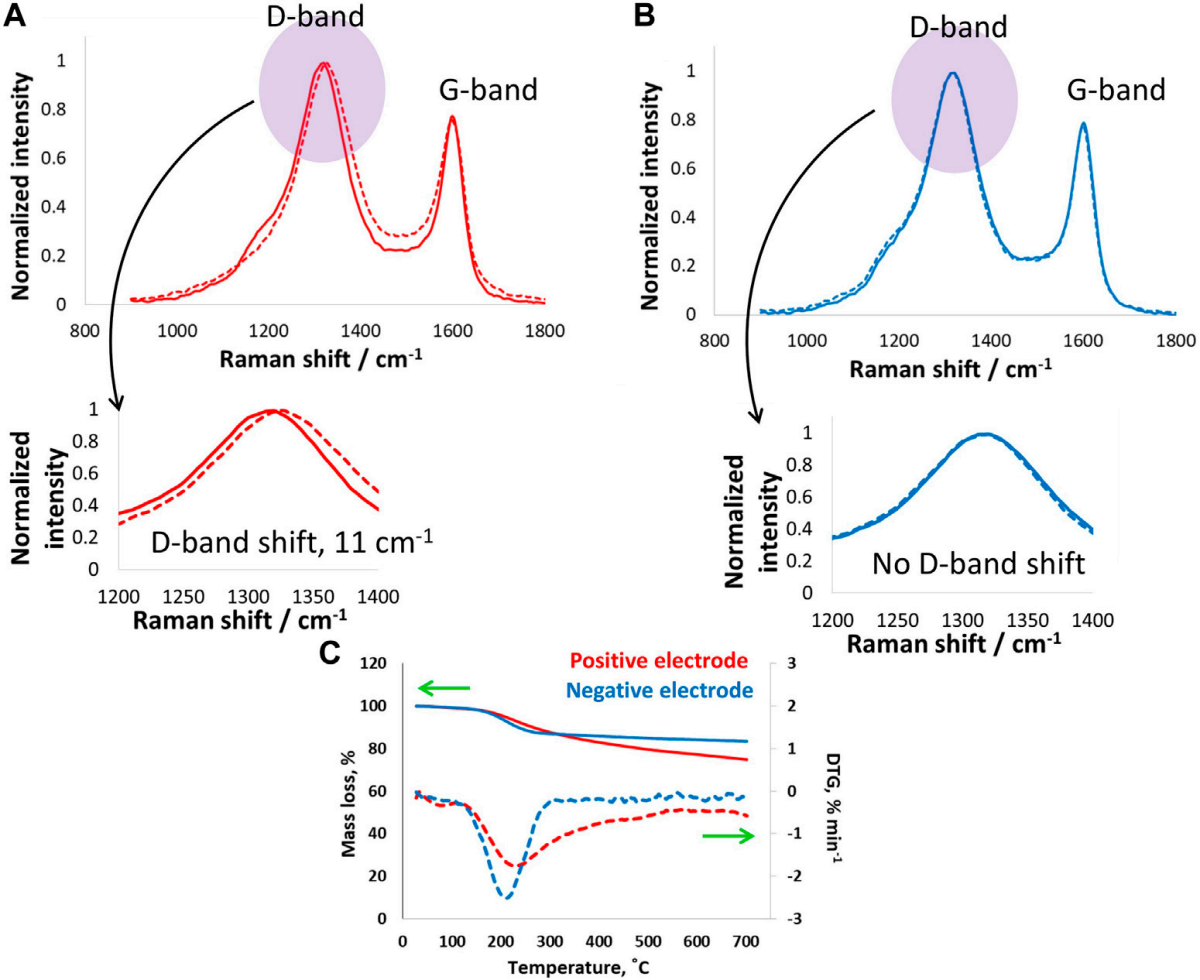
**(A,B)** Raman spectroscopy investigations (D- and G-band) of **(A)** positive and **(B)** negative carbon electrodes after electrochemical tests and floating of the supercapacitor cell at 1.5 V for 100 h at room temperature, and **(C)** thermogravimetry tests on electrodes after floating up to 1.5 V at 60°C.

Postmortem analysis of carbon electrodes by Raman spectroscopy and thermogravimetry is presented in Figure 8. The positive electrode exhibited a D-band shift of 11 cm^−1^, which is higher than the shift for electrodes operated at 24°C and clearly more than those working down to −40°C. The significantly high D-band shift for electrodes operating at 60°C suggests a combined effect of floating at 1.5 V and high temperature causing severe surface changes. Even at such extreme operation conditions, the G-band remained unchanged, as no shift was observed, which suggests no structural changes in the positive carbon electrode. On the other hand, the negative electrode shows no shifts in the D- or G-bands, which confirms its stability because it only underwent hydrogen adsorption and desorption, which are less severe and highly reversible. Overall, the positive electrode capacitance decreased by 23% after floating, and the resistance increased by 48% after floating at 1.5 V for 100 h. These findings are further confirmed by the TG and DTA data in Figure 8C, where higher mass loss was observed for the positive carbon electrodes over a wide temperature range of up to 700°C. On the other hand, the negative electrode showed a sudden mass at approximately 250°C, and then the mass remained more or less constant. The negative electrode mass loss could be due to the formation of weak acidic groups upon interactions with the electrolyte. By contrast, the positive electrode exhibited mass loss in a wide temperature range, which suggests the presence of oxygenated surface functional groups of different natures, such as carbonyl and quinones.

Although the temperature effects of carbon/carbon supercapacitors have been studied in the past, no influence in a wide temperature window has been reported in choline-based aqueous electrolytes. As the negative electrode behavior remained unchanged during harsh experimental conditions, one can attribute these excellent performance parameters to strong hydrogen bonding within the choline and methanol, which restricted electrolyte degradation and to extensive electrolyte reduction.

## 4 Conclusion

Promoted hydrogen bonding between water, methanol, and choline cations, confirmed by Raman spectroscopy, improved the physicochemical properties of electrolytes, which influenced supercapacitor performance. Strong hydrogen bonding enabled the consistent operation of supercapacitors with no aging effects at low temperatures by preventing electrolytes from freezing. It also improved the high-temperature performance where water–methanol interactions led to a relatively high overpotential for hydrogen evolution at the negative electrode. Hydrogen evolution at −40°C at the negative electrode was significantly quenched, which caused the charges to be stored at the EDL without the involvement of Faradaic processes, thus maintaining high energy efficiency values and stable performance during voltage hold tests. The risk of electrolyte chemical reactions with cell components such as carbon electrodes, electrolyte degradation, or with current collectors at high temperatures was reduced by the stability of electrolytes achieved with this composition. As a result, the supercapacitor exhibited high capacitance, long cycle life, and stable performance at both the positive and negative electrodes. The next steps in this research direction include the use of a methanol additive in a choline iodide-based redox-active electrolyte in hybrid supercapacitors and battery electrode testing at low and high temperatures.

## Data Availability

The original contributions presented in the study are included in the article/Supplementary Material; further inquiries can be directed to the corresponding author.
